# Commentary: Basophil Activation-Dependent Autoantibody and Interleukin-17 Production Exacerbate Systemic Lupus Erythematosus

**DOI:** 10.3389/fimmu.2017.00787

**Published:** 2017-07-07

**Authors:** Dimitri Poddighe, Gian Luigi Marseglia

**Affiliations:** ^1^Department of Pediatrics, ASST Melegnano e Martesana, Milano, Italy; ^2^Department of Pediatrics, University of Pavia, Pavia, Italy

**Keywords:** basophils, immunoglobulin E, systemic lupus erythematosus, Th2 response, *Mycoplasma pneumonia*-related extra-pulmonary diseases

Basophils are the least abundant leukocytes in blood, and their biological role has been ignored for long time; moreover, their paucity and the experimental difficulties in studying their function caused an under-estimation of their biological importance until few years ago. In the early 2000s, basophils were recognized as being important cells for host immunity against parasites: indeed, murine models of these intestinal infections had been the main experimental setting to explore basophil biology for several years. Subsequently, the potential role of basophils in the immunity could be much more appreciated, thanks to the development of murine models where basophils were selectively lacking. Then, basophils have been implicated in several allergic diseases and, more generally, in the regulation of Th2 adaptive immune responses. Recently, basophils have been claimed even in the immune-pathogenesis of an autoimmune disease, namely, systemic lupus erythematosus (SLE) (1).

The recent paper by Pan et al. highlighted the potential and emerging role of basophils in SLE and, specifically, in the production of autoantibody, which has been demonstrated to cause some tissue lesions in SLE patients as well as in related animal experimental models. The authors moved from the clinical observation that patients with SLE showed a higher expression of activation markers (CD203c and CD63) on basophils and higher titers of total and auto-reactive IgE (including ANA and anti-dsDNA autoantibody). Therefore, they tested the hypothesis that the activation of basophils may promote the development of SLE in the experimental model represented by lupus-prone MRL-*lpr/lpr* mouse. Interestingly, basophil-depleted MRL-*lpr/lpr* mice exhibited: (i) a prolonged survival; (ii) a decreased level of serum anti-nuclear IgG and IgE; (iii) a reduction of kidney injury due to a lesser deposition of IgG and IgE immune complexes in the glomeruli; (iv) a reduction of IL-17 production (2). Basically, basophil depletion mitigated the clinical expression of SLE-like disease in MRL-*lpr/lpr* mouse. Probably, that result derived from the impaired switch of lymphocytes toward a Th2 phenotype, which promotes the production of some IgG subclasses and IgE and, as a consequence, of SLE-related autoantibody belonging to these isotypes.

After all, previous studies on murine models of allergic asthma, being a typical Th2-driven and IgE-mediated disease, showed a role of basophils as a starting component of the adaptive immune response: here, basophil depletion resulted in the impaired development of IL-4-producing Th2 lymphocytes and, as a consequence, in the reduction of total and specific IgE production (3). Going back to autoimmunity and SLE, the results obtained by Pan et al. strengthened the experimental observations previously made in *Lyn−/−* mice. This mouse being deficient of *Lyn* tyrosine kinase expresses a constitutive Th2 skewing of the adaptive immune response, due to the enhancement of basophil proliferation and of IL-4 production. Importantly, *Lyn−/−* mouse showed also a prominent predisposition to autoimmunity, developing several pathological features that mimic human SLE, such as the nephritis mediated by immune complexes and the production of ANA and anti-dsDNA autoantibody. Previously, Charles et al. described that basophils and basophil-dependent (self-reactive) IgE production were fundamental components for the development of SLE-like disease in this murine model (4).

Therefore, all these studies may provide an overall pathogenic model explaining the coexistence of atopy and autoimmunity in SLE. On the contrary, several autoimmune diseases, such as rheumatoid arthritis, multiple sclerosis, and type I diabetes mellitus, showed an inverse relationship with atopy, but those differ substantially from SLE, as regards both clinical and pathological aspects (5). Actually, the immune-pathological aspects of SLE might be extended to the pathogenesis of more similar immune-mediated diseases, whose mechanisms have yet to be unveiled. For instance, recently we observed elevated levels of serum IgE, namely atopy, in children developing different forms of *Mycoplasma pneumoniae*-related extra-pulmonary diseases (MpEPDs) (6) and we confirmed such an observation in a larger cohort of children, comparing them with patients affected with isolated respiratory diseases (unpublished data). MpEPDs have been supposed to be immune-mediated, but the mechanisms are still almost completely elusive. As well as SLE, MpEPDs can involve multiple organs, including joints, muscles, skin, kidneys, nervous system, as the most affected ones (7). Several studies showed that *M. pneumoniae* infection is able to elicit an IgE response (8), and it is one of the most common agents causing post-infectious acute or recurrent urticarial rashes (9). Thus, an individual with atopic predisposition could have an additional stimulation to produce IgE during *M. pneumoniae* infection; as a consequence, we could speculate that this individual might develop some self-reactive IgE that promotes immune-mediated diseases and manifestations, being different according to the autoantibody cross-reactivity.

So far, there are no available data to firmly support this perspective, but emerging evidences from the aforementioned studies may broaden this possibility to other immune-mediated diseases, in addition to SLE. Although specific data about the effect of *M. pneumoniae* on basophil homeostasis in humans and mice are not available at all, some insights on related immunologic aspects have been recently provided. A paper by Medina et al. reported that a toxin produced by *M. pneumoniae*, named CARDS toxin, is able to increase total serum IgE in BALB/c mice; however, the reactivity of IgE to antigens other than CARDS toxin remains unknown in these animals (10). Another study showed that *M. pneumoniae* was able to enhance the Th17 cell response both *in vivo* and *in vitro* in BALB/c mice, which suggests that it might promote the development of MpEPDs (11). As a human counterpart, serum levels of IL-17 in atopic children with *M. pneumoniae* infection resulted to be significantly increased than in non-atopic controls (12).

In conclusion, the research by Pan et al. highlighted two potential and coexisting immune mechanisms of autoimmunity, namely basophil-dependent (self-reactive) IgE and IL-17 production, which might be implicated also in other autoimmune diseases, in addition to SLE.

## Author Contributions

DP conceived, drafted, and wrote this manuscript; GM provided important intellectual contribution.

## Conflict of Interest Statement

The authors declare that the research was conducted in the absence of any commercial or financial relationships that could be construed as a potential conflict of interest.
